# Antimicrobial and Antioxidant Activities and Phenolic Profile of *Eucalyptus globulus* Labill. and *Corymbia ficifolia* (F. Muell.) K.D. Hill & L.A.S. Johnson Leaves

**DOI:** 10.3390/molecules20034720

**Published:** 2015-03-16

**Authors:** Ștefan Dezsi, Alexandru Sabin Bădărău, Cristina Bischin, Dan Cristian Vodnar, Radu Silaghi-Dumitrescu, Ana-Maria Gheldiu, Andrei Mocan, Laurian Vlase

**Affiliations:** 1 Faculty of Geography, Babeș-Bolyai University, 5-7, Clinicilor Street, Cluj-Napoca 400006, Romania; E-Mail: stefan@geografie.ubbcluj.ro; 2 Faculty of Environmental Sciences and Engineering, Babeș-Bolyai University, 30, Fântânele Street, Cluj-Napoca 400294, Romania; E-Mail: alexandru@transsilvanica.net; 3 Faculty of Chemistry and Chemical Engineering, Babeș-Bolyai University, 11, A. Janos Street, Cluj-Napoca 400028, Romania; E-Mails: cristina_bischin@yahoo.com (C.B.); rsilaghi@chem.ubbcluj.ro (R.S.-D.); 4 Faculty of Food Science and Technology, University of Agricultural Sciences and Veterinary Medicine, 3-5, Manăştur Street, Cluj-Napoca 400372, Romania; E-Mail: dan.vodnar@usamvcluj.ro; 5 Faculty of Pharmacy, Iuliu Hatieganu University of Medicine and Pharmacy, 8, V. Babes Street, Cluj-Napoca 400010, Romania; E-Mail: Gheldiu.Ana@umfcluj.ro

**Keywords:** *Eucalyptus globulus*, *Corymbia ficifolia*, antimicrobial, antioxidants, phenolics

## Abstract

This study was performed to evaluate the *in vitro* antimicrobial and antioxidant activities and the phenolic profile of *Eucalytus globulus* Labill. and *Corymbia ficifolia* (F. Muell.) K.D. Hill & L.A.S. Johnson leaves. Both leave extracts contain significant amounts of phenolic compounds, mainly flavonoids. Qualitative and quantitative analyses of the phenolic compounds were performed using a HPLC/MS method. The main flavonoid was hyperoside and its highest amount was found in *E. globulus* (666.42 ± 5.02 μg/g dw plant material). Regarding the flavonol profile, myricetin was the dominant compound and its highest amount was found in *C. ficifolia* leaves (124.46 ± 0.24 μg/g dw plant material). The antioxidant activity was evaluated by DPPH, TEAC, hemoglobin ascorbate peroxidase activity inhibition (HAPX) and inhibition of lipid peroxidation catalyzed by cytochrome c assays, revealing an important antioxidant potential for both species. In the antimicrobial assays, *C. ficifolia* extract was found to be more active than *E. globulus* against both Gram-positive and Gram-negative bacterial strains with the exception of *Bacillus subtilis*. The results of the present study provide new valuable data regarding the bioactivities of these medicinal species.

## 1. Introduction

Historically, the human pharmaceutical arsenal is significantly indebted to Nature and in particular to natural products obtained of traditional medicinal plants, fungi and bacteria [1]. To date, natural products and compounds derived therefrom command a substantial market share, comprising 61% of anticancer compounds and 49% of anti-infectives approved in the past 30 years [2]. In medicinal plants, these compounds are considered as bioactive natural products and may ultimately be developed as drugs. In food, they would be defined as phytonutrients without therapeutic claims but with significant health benefits that can be used in disease prevention [3].

Under the name of “Gum trees” or “Eucalypts” there are included, from a taxonomic point of view, three genera, namely *Eucalyptus* L’Heritier (1789), *Angophora* Cavanilles (1797) and *Corymbia* Hill & Johnson (1995) [4,5]. The genus Eucalyptus *s.str.* encompasses more than 500 species native mostly to Australia with some species also native in New Guinea and the Philippines [5]. One of the best known species is *Eucalyptus globulus* Labill. or “Blue Gum” which is native to south-eastern Australia and Tasmania [5], but also widely cultivated in the Mediterranean region [6]. Its leaves have been used as traditional remedies for treatment of various diseases such as pulmonary tuberculosis, influenza, fungal infections and diabetes [7,8,9,10]. Because of their antioxidant activity, leaf extracts of *E. globulus* have been also used as food additives [9].

The genus *Corymbia* consists of 113 species which are generally named “bloodwoods” due to their propensity to exude a red gum from the cut bark. All of them are endemic to Australia and can be distinguished from the related genera by their corymb inflorescences [11]. The most well-known and the most cultivated species of the genus is *Corymbia ficifolia* (F. Muell.) Hill et Johnson (previously known as *Eucalyptus ficifolia*) despite the fact that it is a local endemic of the Walpole region of southern part of Western Australia [12]. Even though *Corymbia* species have been mostly used as ornamental trees, recent research has suggested medicinal usage possibilities [13].

Most of the research conducted on *Eucalyptus* has mainly focused on the composition and biological activities of the essential oil extracted from the leaves, revealing important antibacterial properties [6,7,10,14]. However there are some isolated studies that mainly deal with the chemical composition of the leaf extracts and their antimicrobial and antioxidant properties [7,15]. Literature data on *Corymbia* species consists of just a few reports that describe the chemical composition of the leaf essential oil from *Corymbia citriodora* Hook. or other species [16,17] or the composition of fruit resins and their antibacterial properties [13,18], but no data was found regarding *C. ficifolia*. Nevertheless, recent studies on *Eucalyptus* and *Corymbia* report a positive correlation between the antioxidant and antimicrobial properties and the polyphenolic content of stumps and fruits [8,9,13,19]. The importance of phenolic compounds has increased due to the linkage between oxidative stress and the development of chronic diseases, including coronary heart disease, cancer as well as aging. Recently, phenolics have been considered powerful *in vitro* antioxidants and proved to be more potent antioxidants than vitamins C and E and the carotenoids [20]. In this context, further comprehensive studies on polyphenolic compounds and their amounts in plants are essential. A rapid and sensitive HPLC-UV method assisted by mass spectrometry detection was utilized for the determination and quantification of polyphenols in the leaves extracts of these species. The overall aim of this paper was to conduct a comparative study of the chemical composition of *E. globulus* and *C. ficifolia* leaves and to evaluate their antioxidant and antimicrobial activities, for a better usage and characterization of these medicinal herbs.

## 2. Results and Discussion

### 2.1. HPLC-UV-MS Analysis of Polyphenols 

The employed method was developed for the identification and quantification of nineteen phenolic compounds: eight phenolic acids and eleven flavonoids. The quantitative determination was performed by the external standard method and the concentrations of the identified phenolic compounds are shown in Table 1 in order of their retention times.

**Table 1:** The polyphenolic compounds content in the natural products (μg/g dw plant material).

Polyphenolic Compounds	*m/z* Value	R_T_ ± SD (min)	*E. globulus* Leaves	*C. ficifolia* Leaves
Gentisic acid	153	3.69 ± 0.03	NF	<0.02
Chlorogenic acid	353	6.43 ± 0.05	<0.02	<0.02
*p*-Coumaric acid	163	9.48 ± 0.08	NF	<0.02
Hyperoside	463	19.32 ± 0.12	666.42 ± 5.02	454.95 ± 0.02
Isoquercitrin	463	20.29 ± 0.10	38.95 ± 5.72	71.31 ± 0.07
Rutin	609	20.76 ± 0.15	48.65 ± 3.32	180.77 ± 0.21
Myricetin	317	21.13 ± 0.12	92.34 ±0.21	124.46 ± 0.24
Quercitrin	447	23.64 ± 0.13	287.83 ± 2.12	334.57 ± 0.35
Quercetin	301	27.55 ± 0.15	2.01 ± 0.06	5.04 ± 0.04
Luteoline	285	29.64 ± 0.19	34.40 ± 1.73	69.67 ± 0.09
Apigenine	279	39.45 ± 0.15	2.85 ± 0.02	10.69 ± 0.01

Notes: NF—not found, below limit of detection; Values are the mean ± SD (n = 3).

Gentisic, chlorogenic and *p*-coumaric acids were identified in the *C. ficifolia* extract, but none of these compounds could be quantified. The only phenolic acid present in the *E. globulus* extract was chlorogenic acid. 

The patterns of flavonoids and flavonols indicate large quantitative differences. The dominant flavonoid in both extracts is hyperoside, with its highest amount being found in *E. globulus* (666.42 ± 5.02 μg/g dw plant material). Isoquercitrin, rutin and quercitrin were found in both extracts and the highest amount was seen in *C. ficifolia* (71.31 ± 0.07, 180.77 ± 0.21 and 334.57 ± 0.35 μg/g dw plant material, respectively). 

Regarding the flavonol profile, myricetin is the main compound in both extracts, and it is found in a higher quantity in *C. ficifolia* (124.46 ± 0.24 μg/g dw plant material). The flavonol with the lowest amount in both extracts is quercetin (Table 1), the richest species also being *C. ficifolia* (5.04 ± 0.04 μg/g dw plant material). Pereira *et al.* reported higher quantities of quercetin and luteolin (206.51 ± 31.11 and 161.57 ± 24.25 μg/g dw plant material, respectively) in a 70% methanol-aqueous extract [7].

The polyphenols from *E. globulus* and *C.* ficifolia leaves were analyzed. In summary, the simultaneous determination of a wide range of polyphenolic compounds was performed using a rapid, highly accurate and sensitive HPLC method assisted by mass spectrometry detection, and the comparative study showed large quantitative differences between the two species.

### 2.2. Determination of Phenolic Compounds Content

Phenolic compounds are often associated with various positive health effects, including antioxidant effects, decreases in the risk of cardiovascular diseases, anti-cancer mechanisms and anti-inflammatory properties [7,20].

The results of the total phenolic and total flavonoids content of *E. globulus* and *C. ficifolia* extracts are given in Table 2. The total phenolic content (TPC) was expressed as gallic acid equivalents (mg GAE/g dw plant material) and the calculation of total flavonoid content by using a standard curve of rutin and presented as rutin equivalents (mg RE/g dw plant material).

**Table 2:** The content of total polyphenols and flavonoids in the extracts.

Samples	TPC (mg GAE/g Plant Material)	Flavonoids (mg RE/g Plant Material)
*E. globulus* leaves	235.87 ± 4.38	35.76 ± 0.95
*C. ficifolia* leaves	108.51 ± 1.43	44.44 ± 1.23

Notes: Each value is the mean ± SD of three independent measurements; TPC: Total polyphenols content; GAE: Gallic acid equivalents; RE: rutin equivalents.

The leaves of *E. globulus* contain an important amount of phenolic compounds (235.87 ± 4.38 mg/g dw plant material). The content of total phenolics in *C. ficifolia* was significantly lower (108.51 ± 1.43 mg/g dw plant material) but on the other hand higher, regarding the flavonoid content (44.44 ± 1.23 mg/g dw plant material). Regarding the total phenolics content of *E. globulus* leaves extract Pereira *et al.* reported a significantly lower amount of phenolic compounds (62.10 ± 2.49 mg/g dw plant material) in a 70% methanolic-aqueous extract [8]. Hassine *et al.* found a significantly lower amount of phenolics in an ethanolic extract of *E. gillii* (143.4 ± 0.1 mg/g dw plant material) but a comparable amount of flavonoids in the same extract (34.3 ± 0.1 mg/g dw plant material) [21]. The results of this study are also comparable with those of Luis *et al.* who found a total phenolic content of 218.67 ± 4.52 mg/g dw plant material in an 75% ethanolic wood extract of *E. globulus* [6]. No previous data were found regarding the total phenolics or flavonoid content in *C. ficifolia* extracts. The presence of biologically active compounds is regulated by a number of factors, including plant species, genetic factors, geographical location, type of soil, season of harvesting, herb preparation, drying and storage [22].

### 2.3. Antioxidant Activity Assays

The antioxidant potential of *E. globulus* and *C. ficifolia* extracts was tested through the DPPH bleaching method, Trolox equivalent antioxidant capacity (TEAC) assay, hemoglobin ascorbate peroxidase activity inhibition (HAPX) assay and the inhibition of lipid peroxidation catalyzed by cytochrome *c* assay.

The results obtained by the DPPH assay are presented as quercetin equivalents (Table 3). According to this data, the *E. glubulus* leaves extract exhibited a higher antioxidant activity than *C. ficifolia*. In this case, the percentage of DPPH consumption was converted to quercetin equivalents by using a calibration curve (R^2^ = 0.998) with quercetin solutions of 0–12 μM. The higher the rate of DPPH consumption is, the more powerful the antioxidant potential [22,23].

**Table 3:** Antioxidant capacity parameters obtained using several methods for studied samples.

Samples	DPPH (µg QE/mg Plant Material)	TEAC (µg TE/mg Plant Material)	HAPX (%)
*E. globulus* leaves	15.27 ± 1.77	9.02 ± 0.26	61.16 ± 5.56
*C. ficifolia* leaves	11.76 ± 0.71	5.45 ± 0.26	158.20 ± 29.48

Notes: Each value is the mean ± SD of three independent measurements; QE: Quercetin equivalents; TE: Trolox equivalents.

The TEAC results are in agreement with the DPPH values and also correlated with the results obtained by using the other methods. DPPH and TEAC assays are both based on the same principle (free radical scavenging by electron transfer mechanism) and use synthetic radicals which react directly with antioxidants to quantify the antioxidant capacity of the sample, the only notable difference is that in the case of the TEAC and HAPX, the solution is aqueous rather than ethanolic.

The enzymatic antioxidant assay (HAPX) measures the ability of the extract bioactive compounds to quench the damage inflicted by hydrogen peroxide upon hemoglobin. Additional information is provided by this assay, since it involves the interaction between antioxidant molecules with a protein, the ferryl hemoglobin species (resulting from the action of hydrogen peroxide on ferric hemoglobin) [24,25]. As one can see in the results of this study, the HAPX assay does not correlate with the DPPH and TEAC assays and indicates *C. ficifolia* extract as having a superior antioxidant activity. On the other hand, one can suppose that this result suggests a link between the higher content of flavonoids in *C. ficifolia* and the results from this assay.

**Figure 1:**
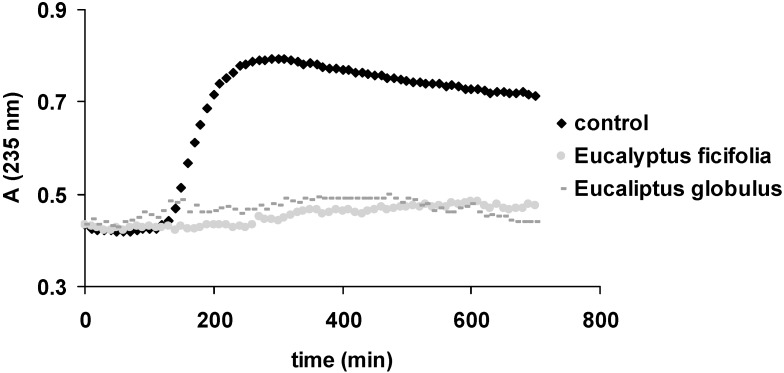
Liposome oxidation by cytochrome *c*, in the presence of the tested samples.

A more physiological method based on peroxidase activity of cytochrome *c* was also assessed to evaluate the antioxidant activity of the extracts. This process monitors the formation of conjugated lipid dienes at 235 nm. According to Yang *et al.*, the inhibition of lipid peroxidation increases with increasing concentrations of flavonoids [26]. Thus, the antioxidant capacity of the tested extracts, reflected in the delay of lipid oxidation, is considered to be based on the same mechanism found in HAPX: the interaction of antioxidants with ferryl hemoglobin, generated in this case by cytochrome *c* [25,27]. In this assay both extracts blocked the process of lipid peroxdation for the whole duration of the whole experiment (700 min), as can be seen in Figure 1.

The antioxidant potential *E globulus* and *C. ficifolia* leaves extracts was investigated using four different methods; the most frequent DPPH and TEAC assays and two complex and physiologically relevant methods based on peroxidase activity of hemoglobin and cytochrome *c*. As one can notice the antioxidant activity of vegetal extracts is strongly related with their chemical composition. As a peculiarity, these extracts contain important amounts of flavonoids and flavonols. High concentrations of flavonoids and flavonols are reflected in the significant scavenging properties [26,28]. There was a significant statistical difference between the analyzed extracts in the DPPH and HAPX assays (0.001 < *p* < 0.05) and a highly significant difference in TEAC method (*p* < 0.001).

### 2.4. Antimicrobial Activity Assays

Plants are important sources of potentially useful structures for the development of new chemotherapeutic agents. The first step towards this goal is the assessment of their *in vitro* antibacterial activity [29]. The *in vitro* antibacterial potential of *E. globulus* and *C. ficifolia* extracts against both Gram-positive and Gram-negative bacteria is summarized in Table 4. As can be seen the *E. globulus* and *C. ficifolia* extracts showed important *in vitro* antibacterial activity against *Staphylococcus aureus* and *Listeria monocytogenes*. The results obtained in the present study revealed that *C. ficifolia* extract was more active than *E. globulus* against both Gram-positive and Gram-negative bacterial strains, with the exception of *Bacillus subtilis*. The best antibacterial activity was shown by both extracts against *Listeria monocytogenes*.

**Table 4:** Antibacterial activity of *E. globulus* and *C. ficifolia* extracts and antibiotic against bacterial species tested by disc diffusion assay.

Bacterial Strains	Standard Antibiotic		Inhibition Zone (mm)	
	Gentamicin	Ciprofloxacine	*E. globulus* Leaves	*C. ficifolia* Leaves
*Staphylococcus aureus*	5.1 ± 0.2	5.2 ± 0.3	8.1 ± 0.1	12.4 ± 0.3
*Bacillus subtilis*	4.2 ± 0.3	4.4 ± 0.1	2.2 ± 0.2	1.1 ± 0.2
*Listeria monocytogenes*	7.3 ± 0.3	6.2 ± 0.2	10.1 ± 0.4	10.5 ± 0.4
*Escherichia coli*	4.1 ± 0.2	5.1 ± 0.4	2.3 ± 0.1	7.3 ± 0.1
*Salmonella typhimurium*	4.2 ± 0.1	4.3 ± 0.1	0	3.2 ± 0.5

Note: Each value is the mean ± SD of three independent measurements.

The strain of *Escherichia coli* was also sensitive to the *C. ficifolia* extract with an inhibition zone of 7.3 ± 0.1 mm of diameter, higher that the inhibition zones of the two standard antibiotics under the present experimental conditions. The results obtained from the antimicrobial properties suggest that *C. ficifolia* is a source of antibiotics inhibiting microbial growth.

The MIC values obtained from antimicrobial tests ranged from 20 to >100 µg/mL (Table 5). The results showed that the bacterial strain *Staphylococcus aureus* was the most sensitive to *C. ficifolia* extract, with a MIC value of 20 µg/mL. *Listeria monocytogenes* with a MIC value of 30 µg/mL was the most sensitive to *E. globulus* extract. Alternatively, *Bacillus subtilis* and *Salmonella typhimurium* were the least sensitive strains to both extracts, with MIC values >100 µg/mL. According to Salvat *et al* plant extracts with MICs of less than/or around 0.5 mg/mL indicate good antibacterial activity. Accordingly, *E. globulus* and *C. ficifolia* extracts exhibited good antimicrobial activity against most of the tested microorganisms [30].

**Table 5:** Minimal Inhibitory Concentration (MIC) of *E. globulus* and *C. ficifolia* extracts.

Bacterial Strains	MIC (µg/mL)	
	*E. globulus* Leaves	*C. ficifolia* Leaves
*Staphylococcus aureus*	50	20
*Bacillus subtilis*	>100	>100
*Listeria monocytogenes*	30	30
*Escherichia coli*	>100	50
*Salmonella typhimurium*	>100	>100

Note: Each value is the mean ± SD of three independent measurements.

## 3. Experimental Section

### 3.1. Plant Materials and Extraction Procedure

The vegetal material from *E. globulus* and *C. ficifolia* leaves was harvested from Perth National Park, Australia, in the summer of 2014. Voucher specimens (Voucher Nos. 390 and 391) were deposited in the Herbarium of the Environmental Science Department, Faculty of Environment Science and Engineering, Cluj-Napoca, Romania. The plant material was air dried at room temperature in the shade, separated and ground to a fine (≤300 µm) powder and then extracted. One gram of each sample was weighed and extracted with 10 mL of 70% ethanol, for 60 min in a sonicating bath at 60 °C. The samples were then cooled down and centrifuged at 4000 rpm for 30 min, and then the supernatant was recovered.

### 3.2. Chemical and Instrumentation

Chlorogenic acid, *p*-coumaric acid, caffeic acid, rutin, apigenin, quercetin, isoquercitrin, quercitrin, hyperoside, kaempferol, myricetol, fisetin from Sigma (St. Louis, MO, USA), ferulic acid, sinapic acid, gentisic acid, gallic acid, patuletin, luteolin from Roth (Karlsruhe, Germany), cichoric acid, caftaric acid were from Dalton (Toronto, ON, Canada). HPLC grade methanol, ethanol, analytical grade orthophosphoric acid, hydrochloric acid and Folin-Ciocalteu reagent were purchased from Merck (Darmstadt, Germany), while hydrogen peroxide, 2,2'-azinobis-3-ethylbenzothiazoline-6-sulphonic acid (ABTS), sodium molybdate dihydrate, sodium nitrite, sodium hydroxide, sodium carbonate, sodium acetate trihydrate, and anhydrous aluminum chloride were from Sigma-Aldrich (Steinheim, Germany). 2,2-Diphenyl-1-picrylhydrazyl (DPPH) and 6-hydroxy-2,5,7,8-tetramethylchroman-2-carboxylic acid (Trolox) were obtained from Alfa-Aesar (Karlsruhe, Germany), HRP (horseradish peroxidase) was purchased from Sigma-Aldrich. Bovine hemoglobin was purified following the general protocol of Antonini and Brunori [31]. The met forms of hemoglobin were prepared by ferricyanide treatment as previously described [32]. Liposomes were obtained by suspending 5 mg/mL soybean lecithin (Alfa Aesar) in phosphate buffer followed by sonication and horse heart purified cytochrome *c* from Sigma-Aldrich [25]. All spectrophotometric data were acquired using a Jasco V-530 UV-VIS spectrophotometer (Jasco International Co., Ltd., Tokyo, Japan).

### 3.3. HPLC-UV/MS Analysis of Polyphenols

#### 3.3.1. Apparatus and Chromatographic Conditions for the Analysis of Polyphenols

The identification and quantification of polyphenolic compounds was carried out using an Agilent Technologies 1100 HPLC Series system (Agilent, Santa Clara, CA, USA) equipped with G1322A degasser, G13311A binary gradient pump, column thermostat, G1313A autosampler and G1316A UV detector. The HPLC system was coupled with an Agilent 1100 mass spectrometer (LC/MSD Ion Trap SL). For the separation, a reverse-phase analytical column was employed (Zorbax SB-C18 100 × 3.0 mm i.d., 3.5 μm particle) and the work temperature was set at 48 °C. The detection of the compounds was performed on both UV and MS mode. The UV detector was set at 330 nm until 17.5 min, then at 370 nm. The MS system operated using an electrospray ion source in the negative mode. ChemStation and DataAnalysis software from Agilent were used for processing the chromatographic data. The mobile phase was a binary gradient: methanol and acetic acid 0.1% (v/v). The elution started with a linear gradient, beginning with 5% methanol and ending at 42% methanol, for 35 min; then 42% methanol for the next 3 min [22,23,33]. The flow rate was 1 mL∙min^−1^ and the injection volume was 5 µL.

The MS signal was used only for qualitative analysis based on the specific mass spectra of each polyphenol. The MS spectra obtained from a standard solution of polyphenols were integrated in a mass spectra library. Later, the MS traces/spectra of the analyzed samples were compared to spectra from library, which allows positive identification of compounds, based on spectral match. The UV trace was used for quantification of identified compounds from MS detection. Using the chromatographic conditions described above, the polyphenols eluted in less than 40 min. Four polyphenols could not be quantified in current chromatographic conditions due to overlapping (caftaric acid with gentisic acid and caffeic acid with chlorogenic acid). However, all four compounds could be selectively identified in MS detection (qualitative analysis) based on differences between their pseudo-molecular mass and MS spectra. For all compounds, the limit of quantification was 0.5 μg/mL, and the limit of detection was 0.1 μg/mL. The detection limits were calculated as minimal concentration producing a reproductive peak with a signal-to-noise ratio greater than three. Quantitative determinations were performed using an external standard method. Calibration curves in the 0.5–50 μg/mL range with good linearity (R^2^ > 0.999) for a five point plot were used to determine the concentration of polyphenols in plant samples [22,23,33,34].

#### 3.3.2. Identification and Quantification of Polyphenols

The detection and quantification of polyphenols was performed in UV-assisted mass spectrometry detection. Due to peak overlapping, four polyphenol-carboxylic acids (caftaric, gentisic, caffeic, chlorogenic) were determined only based on MS spectra, whereas for the rest of the compounds the linearity of the calibration curves was very good (R^2^ > 0.998), with detection limits in the range of 18 ng/mL to 92 ng/mL. The detection limits were calculated as the minimal concentration yielding a reproductible peak with a signal-to-noise ratio greater than three. Quantitative determinations were performed using an external standard method; retention times were determined with a standard deviation ranging from 0.04 min to 0.19 min. For all compounds, the accuracy was between 94.13% and 105.3%. Accuracy was checked by spiking samples with a solution containing each polyphenol in a 10 μg/mL concentration. In all analyzed samples the compounds were identified by comparison of their retention times and recorded electrospray mass spectra with those of standards in the same chromatographic conditions.

### 3.4. Determination of Total Polyphenols and Flavonoids Content

The total phenolic content (TPC) of the extracts was measured using the Folin-Ciocalteu method with some modifications [23,34]. Two mL from each ethanolic extract were diluted 25 times and then mixed with Folin-Ciocalteu reagent (1 mL) and distilled water (10.0 mL) and diluted to 25.0 mL with a 290 g/L solution of sodium carbonate. The samples were incubated in the dark for 30 min. The absorbance was measured at 760 nm, using a JASCO UV-VIS spectrophotometer. Standard curve was prepared by using different concentrations of gallic acid and the absorbances were measured at 760 nm. TPC values were determined using an equation obtained from the calibration curve of gallic acid graph (R^2^ = 0.999). Total polyphenolic content was expressed as mg gallic acid/g dry material plant (mg GAE/g plant material).

The total flavonoids content was calculated and expressed as rutin equivalents after the method described in the Romanian Pharmacopoeia (Xth Edition) [35]. Each extract (5 mL) was mixed with sodium acetate (5.0 mL, 100 g/L), aluminum chloride (3.0 mL, 25 g/L), and made up to 25 mL in a calibrated flask with methanol. Each solution was compared with the same mixture without reagent. The absorbance was measured at 430 nm. The total flavonoids content values were determined using an equation obtained from calibration curve of the rutin graph (R^2^ = 0.999).

### 3.5. In Vitro Antioxidant Activity Assays

#### 3.5.1. DPPH Bleaching Assay

The DPPH assay provides an easy and rapid way to evaluate potential antioxidants. DPPH free radical method is an antioxidant assay based on electron-transfer that produces a violet solution in ethanol. This free radical, stable at room temperature is reduced in the presence of an antioxidant molecule, giving rise to a yellow solution. The free radical scavenging activity of the ethanolic extracts was measured in terms of hydrogen donating or radical scavenging ability using this method. A stock solution of 100 µM DPPH was prepared. In a glass cuvette, 2 µL from original extracts were added to 998 µL DPPH solution. The absorbance changes were monitored at 517 nm for 30 min, using a UV-VIS spectrophotometer equipped with a multi-cell holder. The percentage of DPPH consumption in each case was converted to quercetin equivalents using a calibration curve (R^2^ = 0.991) with quercetin standard solutions of 0–12 µM [23,34]. The higher the rate of DPPH consumption, the more powerful is the antioxidant capacity.

#### 3.5.2. Trolox Equivalent Antioxidant Capacity (TEAC) Assay

In the Trolox equivalent antioxidant capacity (TEAC) assay, the antioxidant capacity is reflected in the ability of the natural extracts to decrease the color, reacting directly with the ABTS radical. The latter was obtained by oxidation of 2,2'-azinobis(3-ethylbenzothiazoline-6-sulfonic acid (ABTS) with peroxide, catalyzed by horseradish peroxidase (HRP). Original extracts were diluted 5 times, and 3 µL from the diluted extract were added to 997 µL ABTS solution. The amount of ABTS radical consumed by the tested compound was measured at 735 nm, after 30 min of reaction time. The evaluation of the antioxidant capacity was obtained using the total change in absorbance at this wavelength. The percentage of ABTS consumption was transformed in Trolox equivalents (TE) using a calibration curve (R^2^ = 0.986) with Trolox standard solutions of 0–16 µM [22,23].

#### 3.5.3. Hemoglobin/Ascorbate Peroxidase Activity Inhibition (HAPX) Assay

Inhibition of hemoglobin ascorbate peroxidase activity assay (HAPX) was conducted according to the procedure described by Mot *et al.* [24]. Hemoglobin was purified according to the Antonini and Brunori protocols [31]. The reaction was triggered by the addition of met hemoglobin (6 µM) to a mixture of ascorbate (160 µM), peroxide (700 µM) and extracts (5 µM) from the stock solution diluted five times, and it was monitored at 405 nm. This method allows us to evaluate the inhibition of ferryl formation by ascorbate in the presence of the tested compounds. An increase in the time of inhibition reflects the antioxidant capacity of the compound, whereas a decrease, indicates a pro-oxidant effect [32].

#### 3.5.4. Inhibition of Lipid Peroxidation Catalyzed by Cytochrome *c*

Liposomes were obtained by suspending 5 mg/mL soybean lecithin in phosphate buffer (20 mM, pH 7), followed by sonication for 15 min in an ultrasonic bath (using a Power Sonic 410 device, Thermoline Scientific, Wetherill Park, NSW, Australia). The liposome oxidation experiment was performed at room temperature, for 700 min, in the presence of cytochrome *c* (2 µM) and extracts (5 µL from the diluted extract) by monitoring the absorbance at 235 nm (wavelength specific for liposome oxidation). This process monitors the formation of lipid conjugated dienes at the specified wavelength [25].

### 3.6. Determination of Antimicrobial Activity

#### 3.6.1. Microorganisms and Culture Growth

The microorganisms used for antimicrobial activity evaluation were obtained from the University of Agricultural Sciences and Veterinary Medicine Cluj Napoca, Romania. The Gram-positive bacteria *Staphylococcus aureus* (ATCC-25923), *Bacillus subtilis* (ATCC-12228), *Listeria monocytogenes* (ATCC-19115) and gram-negative bacteria *Escherichia coli* (ATCC-25922) and *Salmonella typhimurium* (ATCC-14028). The stock cultures of microorganisms used in this study were maintained on plate count agar slants at 4 °C. Inoculum was prepared by suspending a loop full of bacterial cultures into 10 mL of nutrient agar broth and was incubated at 37 °C for 24 h. About 60 µL of bacterial suspensions, adjusted to 10^6^ CFU/mL were taken and poured into Petri plates containing 10 mL sterilized nutrient agar medium. Bacterial suspensions were spread to get a uniform lawn culture [36].

#### 3.6.2. Antimicrobial Activity Assay

Antimicrobial activities of the *E. globulus* and *C. ficifolia* extracts were evaluated by means of agar-well diffusion assay with some modifications [36]. Fifteen millilitres of the molten agar (45 °C) were poured into sterile Petri dishes (Ø 90 mm). Cell suspensions were prepared and 100 µL was evenly spreader onto the surface of the agar plates of Mueller-Hinton agar (Oxoid, Basingstoke, UK). Once the plates had been aseptically dried, 6 mm wells were punched into the agar with a sterile Pasteur pipette. The different extracts (10 mg/mL) were dissolved in dimethylsulfoxide/water (1/9) and 80 µL were placed into the wells and the plates were incubated at 37 °C for 24 h. Gentamicin (25 µL/wells at concentration of 4 µg/mL) and ciprofloxacin (5 µg/mL) were used as positive control for bacteria. Antimicrobial activity was evaluated by measuring the diameter of circular inhibition zones around the well. Tests were performed in triplicate and values are the averages of three replicates [30].

#### 3.6.3. Minimum Inhibitory Concentration

Based on the previous screening the minimum inhibitory concentration (MIC) of both *E. globulus* and *C. ficifolia* extracts was analyzed through the agar-well diffusion method. A bacterial suspension (10^5^–10^6^ CFU/mL) of each tested microorganism was spread on the nutrient agar plate. The wells (6 mm diameter) were cut from agar, and 60 µL of each *E. globulus* and *C. ficifolia* extracts dissolved in dimethyl sulfoxide (DMSO) at different concentrations (10, 20, 25, 50 75 and 100 µg/mL) were delivered into them. The plates were incubated at 37 °C for 24 h under aerobic conditions that followed by the measurement of the diameter of the inhibition zone expressed in millimeter. MIC was taken from the concentration of the lowest dosed well visually showing no growth after 24 h [30].

### 3.7. Statistical Analysis

The experiments were designed and the experimental data evaluated using one-way analysis of variance (ANOVA), with *p* < 0.05 as threshold for statistical significance. The statistical results confirm the hypothesis that the differences between the results are either not significant (*p* > 0.05), significant (0.001 < *p* < 0.05) or highly significant (*p* < 0.001). The average of multiple measurements (triplicates or more) was listed in the tables together with the standard deviations. Statistical analysis was performed using Excel software package.

## 4. Conclusions

The results of the present study reveal important data regarding the phenolic composition, antioxidant and antibacterial activities of the two medicinal species *E. globulus* and *C. ficifolia*. The differences between the two species are mostly quantitative and are related to the respective flavonoid and flavonol profiles. The dominant flavonoid glycoside in both species is hyperoside, with its highest amount being found in *E. globulus*. Regarding the flavonol profile, *C. ficifolia* contains higher amounts in each of the identified compounds. Nevertheless gentisic and *p-*coumaric acids were found just in the *C. ficifolia* extract. The antioxidant potential of the two species was tested through several assays, which indicated both species as valuable sources of free radical scavenging compounds. However, the HAPX assay results don’t correlate with the DPPH and TEAC assays and show *C. ficifolia* extract as having a superior antioxidant activity. This might suggest a link between the higher content of flavonoids in *C. ficifolia* and the results from this assay. Referring to antibacterial assays, *C. ficifolia* extract was found to be more active than *E. globulus* against both Gram-positive and Gram-negative bacterial strains with the exception of *Bacillus subtilis*; the best antibacterial activity was shown against *Listeria monocytogenes*. The MIC results showed that the bacterial strain *Staphylococcus aureus* was the most sensitive to *C. ficifolia* extract and *Listeria monocytogenes* was the most sensitive to *E. globulus* extract.
